# Training-Schools and Business Habits

**Published:** 1919-04-12

**Authors:** 


### THE MATRONS' AND SISTERS' DEPARTMENT.

### TRAINING-SCHOOLS AND BUSINESS HABITS.

There is one deficiency in nurses constantly obtruded upon public notice, and this is lack of business ability. It is true that there are thousands of business women in the ranks, as witness the unexampled prosperity of the Royal National Pension Fund for Nurses. But the rank and file, what may be called average nurses, commonly display in all their dealings with money, and in their dealings with employers, an almost infantile inability to understand either their own interests or their obligations towards others. We have observed the same strange disregard of business precepts, we might almost say of common sense, in members of religious orders, but in their case, since they are vowed to poverty, seldom handle coin and are provided for during their whole lives, it is of less importance. But the nurse is a wage-earner. Her working years are few in comparison with her life as a whole; she is frequently called to offices in which she imperatively needs business habits. It is a grave defect that she should leave her training-school so entirely inexpert in the business ways of the outside world.

We are aware that the training-schools have quite enough necessary knowledge to impart to their nurses in matters relating directly to their calling, and can hardly be expected in all respects to supplement the very inadequate education women now receive. But in the business of turning out capable women for the public service, in equipping their probationers to make a good career as nurses, it is necessary for the authorities to take well into consideration the material as it comes into their hands. A woman of twenty ought undoubtedly by that time to have been properly instructed in money matters, accounts, the obligations of a contract, and other things necessary to enable her to hold her own in the world and give all their due. But as a matter of fact she is seldom found to have received any instruction at all in these things. Unless during her training she learns the things which belong to her status as an independent woman she will not learn them at all, and never having learned them will never be more than an imperfect nurse.

The new Education Act gives ground for hope that the kind of instruction we have indicated may come in time to be reckoned an essential feature in the education provided by the State in elementary and secondary schools. But before it comes fully into force several generations of probationers will have passed through the training-schools. It will be regrettable if nothing is done to raise the business standard of nurses in the meantime. With the introduction of the Sister Tutor, efforts in the direction of repairing neglected education in nursing students are now possible. Would it not be feasible to graft on to the probationers' course some instruction which might open their eyes to their duties and privileges as regards money?

The recent complaints made by and on behalf of military nurses in respect of their demobilisation grant form an instructive commentary on the unbusinesslike notions of some nurses in connection with money matters. There are two ways of effecting demobilisation; the one to grant a month's pay with allowance for board, and this is the system adopted for the rank and file of the Army; the other, to confer a gratuity according to length of service, and this is adopted for officers. In the case of an Army nurse, a month's pay with allowance for board would work out at about £10. Her gratuity after four years' service would amount to between £30 and £40. Yet an agitation has been promoted which reckons it a grievance that Army nurses are treated on the latter rather than on the former footing, and this avowedly on the ground that certain nurses, after three or four years of continuous employment with full allowances, have not had the ordinary prudence, while anticipating demobilisation for the last six months, to reserve out of their pay sufficient money to enable them to take a few weeks' holiday in consideration of the gratuity shortly to be placed to their credit. There is no other class of workers, either male or female, on behalf of whom such an extraordinary grievance could have been set on foot. We are speaking now purely of the business aspect of the case. That individuals in authority may have acted in an inconsiderate manner is highly probable, for the War Office is still, we fear, haunted as ever by arbitrary officials.

A great deal is expected from the Nurses' Registration Bill, possibly more than any Bill yet framed could expect to fulfil. Should it become law, and a General Nursing Council proceed to draw up the outlines of a syllabus on which nurses shall hereafter be examined, is it too much to hope that one or two elementary questions testing the candidates' knowledge of simple business propositions may be inserted?

